# Development of a graphene field effect transistor-based immersible biosensor for immunodetection of the birch pollen allergen Bet v 1 in air samples

**DOI:** 10.1016/j.heliyon.2024.e38922

**Published:** 2024-10-04

**Authors:** Stefan Jarić, Mario Wenger, Ivan Bobrinetskiy, André Stapelfeldt, Glorismer Pena-Amelunxen, Branko Šikoparija, Lorenz Aglas

**Affiliations:** a BioSense Institute - Research and Development Institute for Information Technologies in Biosystems, University of Novi Sad, Novi Sad, 21000, Serbia; b Department of Biosciences and Medical Biology, Paris Lodron University of Salzburg, Salzburg, 5020, Austria

**Keywords:** Biosensor, Allergen detection, Birch pollen, Graphene field effect transistor, Air sampling

## Abstract

Pollen traps, the current gold standard to determine pollen load and thereby the allergy season, are not sufficient to determine the allergenic risk. Therefore, the establishment of highly sensitive assays for allergen measurement is of highest interest. Herein, a graphene field-effect transistor (GFET) was constructed on an interdigitated electrodes chip to develop an immersible biosensor, which was used to detect the major birch pollen allergen Bet v 1. Graphene was wet-transferred on interdigitated electrodes that contain a reference electrode used as a liquid gate in the GFET. Using a standard ELISA protocol, two different anti-Bet v 1 antibodies were chosen and immobilized on graphene for the specific capture of the target allergen. The sensitivity of the GFET biosensor was evaluated using a standard Ag/AgCl liquid gate electrode and a reference electrode when the chip was immersed in Bet v 1-containing solutions. The results showed a higher performance and sensitivity for Bet v 1 detection compared to a mediator release method, one of the most sensitive assays for allergen detection. Compared with conventional methods of allergen detection, these immersible biosensors significantly improved the speed and level of detection providing the foundation of a point-of-need platform for in-field application. Furthermore, the proposed technique provides both a new biosensor for allergen detection and a strategy for designing low-cost integrated biosensors.

## Highlights

• Using highly sensitive methods are able to detect specific allergens within air pollen samples.
• A graphene-based biosensor was developed by immobilizing Bet v 1 specific IgG antibodies.
• Bet v 1 detection with the biosensor demonstrated a sensitivity of 10 pg/mL.
• Biosensor has the potential for future in-field allergen detection.

## 1 Introduction

Early flowering wind-pollinated trees, such as hazel and birch, are the primary cause for pollen-induced allergic symptoms in springtime, including hay fever and allergic asthma. The sensitization rates to birch pollen range between 8 and 15 % among the general European population, numbers still rising. Over 96 % of all birch pollen allergic patients are sensitized to the major birch pollen allergen Bet v 1, belonging to the family of pathogenesis-related proteins class 10 (PR-10) [1].

Since symptom severity highly depends on the level of exposure and because allergen avoidance during the pollen season is hardly possible, various pollen monitoring and forecast systems have been developed in order to measure the pollen content in the air [2]. These systems provide a useful guide for allergic individuals in terms of symptom prevention and medication intake. However, especially pollen maps, generated by pollen data sets are partially incomplete and lack accuracy to reflect the actual allergen load [3]. Currently, the amount and nature of pollen collected by so-called pollen traps are only determined by microscopy and no associations between pollen count and the actual allergen content – e.g., allergen concentration increases throughout plant development – are made [4]. Even a high allergenic load can be measured in the absence of pollen grains (e.g., rain, thunderstorms) [5,6]. The measurement of total airborne proteins has been suggested but is also not suitable to monitor specific allergens. As realistic conclusions about real-life allergen exposure cannot be drawn, it is crucial to have a precise measure of the specific allergen concentration in the ambient air combined with the pollen load. Together, this information will highly improve the disease management of allergic patients (e.g., earlier medical intervention) [7]. Therefore, the possibility of real-time detection of pollen allergens in aerosol samples would represent a highly relevant tool in the re-evaluation of allergenicity of pollen exposure and will lead to the development of personalized sensors for dose response evaluation. The detection of specific allergens in pollen sources are usually colorimetric or fluorescence-based antibody-meditated detection systems such as enzyme-linked immunosorbent assays (ELISAs) or Western blots. These methods have disadvantages such as high costs and the need for expansive consumables, a low throughput rate, low sensitivity and the requirement for trained personnel performing the laborious and time-consuming assays. Fast and sensitive diagnostic tools that can easily be applied in the field (i.e., point-of-need), with an understandable readout for non-experts, are necessary in improving the current pollen exposure forecast systems in order to draw conclusions regarding their allergenicity in real-life situations, especially during the birch pollen season.

Biosensors based on carbon-based nanomaterials (such as graphene or semiconducting carbon nanotubes) have been shown to be fast and highly sensitive detection techniques for different analytes, including the egg allergen ovalbumin [8]. The physical properties of graphene owing to a high sensitivity to low charge variations provide a limit of detection (LOD) down to single molecules. Nevertheless, the high sensitivity of sensors based on graphene and related nanomaterials suffers from even the slightest environmental changes, which raises the main issue of non-specific reactions and selectivity to the target analyte. E.g., the complex composition of pollen extracts, containing lipids, carbohydrates, insoluble cell wall components and other proteins besides the allergens, might interfere with the performance of the biosensor. Various antibody- or aptamer-based approaches have been suggested to overcome this problem. The accurate assembly of sensors based on graphene linked to receptor molecules remains a challenging task for specific types of antibodies with regard to proper site positioning of the protein to achieve maximum binding efficiency. This can be solved by functionalization or modification of the properties of graphene to increase the number of antibody binding sites.

In this paper, we present the development of an immersible and disposable graphene field-effect transistor (GFET) immunosensor on commercially available interdigitated gold electrodes (IDEs) and demonstrate its analytical performance in the detection of Bet v 1. The immunosensor was produced by wet transfer of graphene on IDEs, followed by functionalization with a Bet v 1-specific immunoglobulin G (IgG) antibody. The immunosensor performance was evaluated by measuring current-voltage characteristics in solutions with different concentrations of Bet v 1. The results show a high sensitivity of the immersible GFET to Bet v 1, thereby outperforming existing methods for allergen detection with a LOD down to 10 pg/mL.

## 2 Materials and methods

### Ethical statement

For sensitization with patients′ sera and the serum pool, four sera from birch pollen allergic patients, were obtained from the CREATE serum bank [9,10], which is available for researchers, and was kindly provided by Prof. Dr. Ronald van Ree, Amsterdam, the Netherlands. Ethical approval for using human sera was obtained from the institutional review board of all participating centers, and each patient provided written consent within the CREATE project. The ethical approval number for the herein reported data is not required/applicable.

### 2.1 Chemicals, antigens and antibodies

Graphene monolayer on Cu foil was purchased from Graphenea (San Sebastian, Spain). Poly(methyl methacrylate) (PMMA), average Mw ∼350,000, powder was obtained from Sigma Aldrich (USA). Toluene and 2-propanol were obtained from Sigma-Aldrich (USA). Tetrahydrofuran (THF), 99.9 % was purchased from Carl Roth (Germany). Acetone was purchased from Honeywell (Charlotte, USA). Phosphate-buffered saline (PBS), 10X was purchased from Fisher Bioreagents (USA). Monoclonal anti-Bet v 1.0101 and affinity-purified polyclonal rabbit anti-Bet v 1.0101 antibodies were produced in-house (University of Salzburg) [11]. Horseradish peroxidase-conjugated anti-IgG antibody was purchased from Jackson Immuno Research (USA). Recombinant Bet v 1 (isoform 1.0101) was produced in-house (University of Salzburg) [12]. Two different commercially available birch pollen batches (batch 1 and batch 2) were purchased from Allergon AB (Sweden) and aqueous extracts (cBPE) were generated as described previously [13]. KPL SureBlue™ (Sera Care, USA) TMB was used as ELISA substrate. Humanized Rat Basophillic Leukemia cell medium: MEM with Earl's salts without L-Glutamine medium (Sigma-Aldrich, USA), 5 % FCSi (Sigma-Aldrich, USA), 4 mM L-Glutamine (Sigma-Aldrich, USA), and 1 % G418 (Sigma-Aldrich, USA). AG-8 cell medium: OptiMEM I + Glutamax I (Gibco by Lifetechnologies, USA), 1 % PenStrep (Sigma-Aldrich, USA) and 5 % FCSi. Tyrode's salts, sodium bicarbonate, Deuterium Oxide and 4-Methylumbelliferyl-β-D-Glucuronid were all purchased from Sigma-Aldrich (USA).

### 2.2 Fabrication of GFET on IDEs and characterization

Monolayer graphene on copper foil (30 μm thickness) was transferred on IDE G-IDE222 electrodes (Drop Sens, Spain) by the wet transfer approach using the supporting thin film of PMMA. In short, PMMA was spin-coated over the graphene/Cu flake (3 × 3 mm) and pre-baked at 60 °C for 5 min. The Cu foil etching was initiated in aqueous mixture of hydrochloric acid, hydrogen peroxide, and water (1:2:20 v/v) for 3 min, and immediately transferred into 0.1 M ammonium persulfate aqueous solution to etch the Cu foil completely for 2 h. After several water baths to wash away the etchant, graphene/PMMA flake was transferred onto the electrode and left overnight to dry. PMMA was cleaned with boiled acetone (three baths) and boiled THF/water mixture (1:3 v/v) to completely dissolve and remove the PMMA film, then thoroughly rinsed with water and dried with N_2_ gun. Current voltage characteristics (CVC) were measured using Ag/AgCl as a liquid gate electrode. Also, co-planar gold (reference) electrode (RE) was used as a liquid gate electrode for comparison. Such composition of IDE was characterized by Raman spectroscopy (Horiba XploRA Plus Raman Spectrometer, France) using 532 nm laser source with 0.9 mW laser power, a 100X objective (Olympus, Japan) and an NA 0.9, and 1200 grating setup.

### 2.3 Biofunctionalization of the GFET with anti-Bet v 1 antibodies

Two different types of monoclonal anti-Bet v 1 antibodies (mAbs) were selected based on the performance determined via ELISA (see below). Antibodies (mAb1 and mAb2) were immobilized on the GFETs. For this, 5 mM PBASE was deposited onto the zero potential graphene and −0.3 V was applied to the Au auxiliary electrode (AE) and incubated for 2 h. It was washed thoroughly with DMF, IPA, and DI water for 1 min each. The GFET was carefully air-dried with a N_2_ gun. 50 μL of pure antibody solution was incubated on graphene overnight at 4ᵒC; Chip GFET1: antibody mAb1 (1.20 mg/mL) and Chip GFET2: antibody mAb2 (1.01 mg/mL). The graphene/IDE chip was rinsed with 1xPBS. Graphene channel was blocked for non-specific binding using 0.5 % bovine serum albumin (BSA) and 0.05 % Tween for 1 h at room temperature (RT). After all measurements, the transistors were gently rinsed with 1xPBS by pipetting and then in 0.1 M citric acid solution (pH 3) to regenerate the biosensor.

### 2.4 Air pollen sampling and extraction

Air samples were collected at the roof level in Novi Sad, Serbia, using an all glass impinger Biosampler (SKC, USA) on March 29th, 2023 (10:20–11:05 and 14:40–15:50 local time) and March 30th (8:25–9:00 local time). The sampler constantly aspirated 12.5 L/min of air, with particles being captured directly into PBS. The samples were lyophilized and reconstituted in a lower volume of dH2O to concentrate the sample for allergen quantification (8-fold). To further concentrate the sample (13-fold) and decrease salt concentrations, Amicon 3kD filters (Sigma Aldrich, USA) were used and the supernatant was stored in the freezer at −20 °C for further use. The presence of birch airborne pollen during all sampling periods were confirmed in simultaneous side-by-side measurements using Hirst method (EN16868). Initially, the analysis of about 10 % of 2-hourly sample surface did not record birch pollen but the analysis of the entire sample revealed the presence of only very few birch pollen grains: 9 pollen grains on March 29th 10:00–12:00 (corresponding to 8 pollen/m^3^), 4 pollen grains on March 29th 14:00–16:00 (corresponding to 3 pollen/m3) and 2 pollen grains on March 30th 8:00–10:00 (corresponding to less than 2 pollen/m3).

### 2.5 Enzyme-linked immunosorbent assay

Eight different mAbs against Bet v 1, recognizing collectively four distinct epitopes of the allergen were tested using an in-house sandwich ELISA. In brief, Nunc MaxiSorp® 96-well plates (Thermo Fisher Scientific, USA) were coated with 2 μg/mL monoclonal anti-Bet v 1, diluted in 1xPBS overnight at 4 °C. Following blocking the wells with 0.5 % BSA, serial dilutions of recombinant Bet v 1 (start concentration 1 μg/mL) were incubated for 2 h at RT. Affinity-purified polyclonal rabbit anti-Bet v 1 in combination with a horseradish peroxidase-conjugated goat anti-rabbit IgG antibody, recognizing the polyclonal antibody, were used for detection and incubated for 1 h each. As a substrate for development, TMB was used, and the reaction was stopped with 1N HCl. Plates were analyzed at a wavelength of 450 nm using an Infinite 200 Pro plate reader (Tecan, Switzerland). The absorbance values were background-subtracted and triplicates were averaged.

For the quantification of Bet v 1 in different cBPE and air samples, the same approach with a serial dilution (starting concentration 4 μg/mL) or undiluted, respectively, was used. The final Bet v 1 concentrations were calculated by generating a linear regression of the recombinant Bet v 1 standard curve.

### 2.6 huRBL mediator release assay

This *in vitro* method uses humanized rat basophilic leukemia cells (huRBL cells) from the cell line RBL-2H3, which were transfected to express the human Fcε receptor I (FcεRI), the human high affinity receptor for IgE antibodies. The protocol for the huRBL mediator release assay was followed as previously described [14] and is briefly reported here. Before passively sensitizing the cells, the Bet v 1 allergic patient serum's complement system was deactivated by incubating the serum with Ag-8 cells at a 1:10 ratio for 1 h. After Ag-8 incubation, the mixture was centrifuged for 5 min at 250g, and the supernatant was collected and added to the huRBL cells for passive sensitization with IgE overnight. The following day, the cells were stimulated with a 1:10 serial dilution of the Bet v 1 allergen, BPE or the air pollen samples (1 μg/mL, 5 μg/mL start concentration or undiluted, respectively) to induce IgE crosslinking and subsequent mediator release. β-hexosaminidase was used as a surrogate marker for mediator release, and the substrate 4-Methylumbelliferyl b-D-glucuronide dihydrate (4MUG, Sigma, Darmstadt, Germany) was used to measure its presence in the supernatant of huRBL cells. As controls, untreated cells (unsensitised and not presented with antigen) were used. At least two wells were used to measure the background signal, and four for the maximal mediator release, which was produced by lysing the cells with 1 % Triton X-100. The fluorescence of the samples was measured with a Tecan M200 plate reader (excitation wavelength: 360 nm, emission wavelength: 465 nm; optimal gain). After background subtraction, results were expressed as a percentage of the maximal lysis. The MTT assay was performed to determine the viability of the cells after serum sensitization and allergen stimulation. To obtain an optimized RBL protocol for the measurement of biologically relevant, pollen-containing air samples, four different sera containing high levels of Bet v 1-specific IgE were used individually to sensitize the cells. The BPE starting concentration used for cell stimulation was adjusted to the Bet v 1 concentration measured in the sandwich ELISA. The serum with the highest mediator release was used for the measurement of the air pollen sample.

### 2.7 GFET biosensor measurements

The analyte solutions were prepared from recombinant, purified Bet v 1 protein starting from a concentration of 100 ng/mL followed by a 1:10 serial dilution until a concentration of 0.1 pg/mL was reached, to determine the detection limit of the GFET biosensor. The Bet v 1 drop-based detection experiments were conducted as follows. A drop (40 μL) of each concentration was added to the transistor, which was then incubated at RT for 10 min. Then the Ag/AgCl pellet electrode (Science Products GmbH, Germany) was placed in the Bet v 1 solution and the I_d_-V_g_ curve was measured. For comparison, immersible biosensing was done by immersing the biosensor into a reaction tube with the analyte and incubated for 10 min at RT.

To ensure that the mAb had been properly immobilized on the biosensor and was functionally active, the sandwich ELISA protocol was conducted with the biosensor, using a fixed allergen concentration of 1 μg/mL Bet v 1. For the secondary antibody control, the same experiment was performed but without adding the allergen this time. Thereby the biosensor was renatured by covering it three times in 0.1 M citric acid for 5 min with PBS washing steps in between. Following the completion of the washing procedure, the allergen (1 μg/mL) was reapplied to the biosensor for repeating the sandwich ELISA protocol to evaluate the biosensor's reusability.

### 2.8 Statistics

**Fig. 1:**
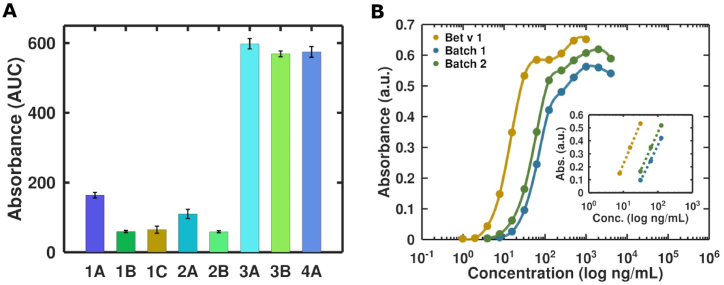
Determination of sensitivity of Bet v 1-specific mAb (1A-C; 2A-B; 3A-B and 4 A). The AUC values of absorbance values of the Bet v 1 titration are shown for simplified representation (titration curves not shown). 1B = mAb1 and 4A = mAb2 were further used in the GFET biosensor **(A)**. Semi-log plot of absorbance versus Bet v 1 concentrations. The assay was used to quantify the Bet v 1 concentration in different batches of birch pollen extracts **(B)**.

**Fig. 2:**
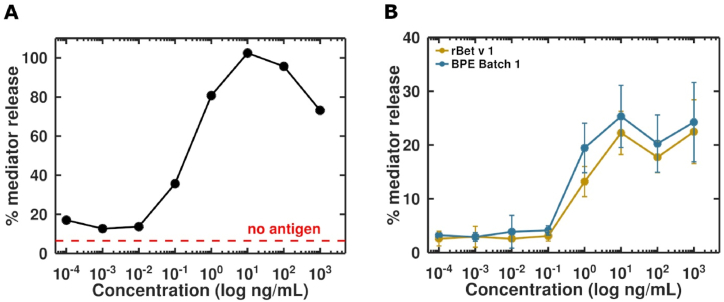
Mediator release curve of Bet v 1-stimulated huRBL cells. The dashed line represents control cells without added antigen **(A)**. Mediator release curve of four different patient sera used for sensitization, illustrated as the median. Sensitized cells were either stimulated with a serial dilution of rBet v 1 or BPE batch 1 **(B)**.

**Fig. 3:**
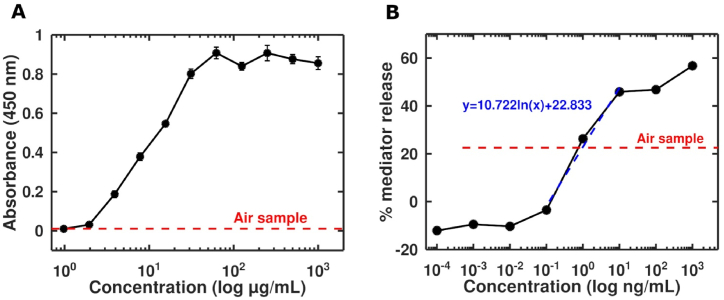
Quantification of Bet v 1 in air samples. The collected air sample was tested in **(A)** ELISA and **(B)** mRBL assay with linear regression for quantification.

Statistical analyses were performed using GraphPad Prism 9. A One-Way ANOVA with Tukey's multiple comparison test was used (Fig. 7). Error bars indicate the standard error of the mean across duplicate or triplicate measurements (Fig. 1, Fig. 2, Fig. 3, Fig. 7). For the Bet v 1 titration, using different mAbs, the area-under-the-curve (AUC) was calculated (baseline Y = 0). The LOD was determined as the background value + 3x the standard deviation of the background values. The significances were displayed with asterisks: p < 0.05 (∗), p < 0.01 (∗∗), p < 0.001 (∗∗∗), and p < 0.0001 (∗∗∗∗).

## 3 Results

### 3.1 Difference of mAb in epitope recognition and sensitivity

To identify the best suitable antibody for our GFET biosensor, the sensitivity of eight mAbs, recognizing four different epitopes of Bet v 1, was tested in an in-house-developed sandwich ELISA. Three of them showed a very high sensitivity (3A, 3B and 4A), whereas the former two recognized the same epitope. The remaining antibodies were moderate in Bet v 1 recognition (Fig. 1A). One antibody with a high (4A) and one with a moderate (1B) sensitivity were considered for further experiments using the GFET biosensor.

In the following, an antibody from the previous experiment with a high affinity for Bet v 1 was used to demonstrate its capability to quantify the Bet v 1 concentration in highly complex biological samples, like birch pollen extracts. The obtained absorbance values were plotted against the logarithm of analyte concentrations (Fig. 1B).

The linear part of the semi-log curve was fitted using the logarithmic fitting to obtain the function of the following type: y = *a*∗**ln**(x) + *b.* By inserting the absorbance value from the linear part of the log fit line the Bet v 1 concentration in the extracts was calculated. Batch 1 of cBPE contained 17 % Bet v 1, while batch 2 had 25 % Bet v 1.

### 3.2 Mediator release assay, a highly sensitive approach to detect Bet v 1

The mediator release assay is a highly sensitive method to determine the allergic immune response occurring upon allergen cross-linking of IgE antibodies on basophils. The results are shown in Fig. 2 and expressed as percentage of the max. lysis value. The huRBL assay in our experiment was about 10 to 1000-fold more sensitive than the sandwich ELISA (Table 1) but varied depending on the sera used for passive sensitization of cells. The maximum mediator release of 100 % in the sensitized cells was reached when stimulating with 10 ng/mL of Bet v 1 (Fig. 2A). The mediator release curve reached the logarithmic increase phase already at a concentration of 0.1 ng/mL with a corresponding mediator release of 40 %. The four tested birch pollen allergic patient's sera show a very similar mediator release with a maximum release at 10 ng/mL Bet v 1 (Fig. 2B). The BPE treated cells followed a similar dose-response curve as cells treated with the Bet v 1 standard, showing the robustness and sensitivity of this approach in detecting Bet v 1 even in highly complex solutions like aqueous pollen extracts.

**Table 1:** Comparison of GFET biosensor with conventional assays for Bet v 1 detection.

Method	LOD	Assay time	Complexity
ELISA	1–5 ng/mL	2 d	Need for skilled personnel and equipped laboratory
Mediator release	30–100 pg/mL	2 d	Need for skilled personnel and equipped laboratory
GFET biosensor	10 pg/mL	12 min	Simple use of the sensor and possibility for in-field application

### 3.3 Air samples

To quantify Bet v 1 in in-field collected air pollen samples, the sandwich ELISA and the mediator release assay protocol were used. For the RBL assay, an average Bet v 1 concentration of 0.785 ng/mL was calculated for the air sample, measured in duplicates. In contrast to the mediator release assay, Bet v 1 was under the detection limit in the sandwich ELISA (Fig. 3A and B). In conclusion, the sensitivity of the sandwich ELISA is not high enough to quantify Bet v 1 in air pollen samples and is thereby not suitable to detect allergens in low quantities which are still detectable by the RBL assay.

### 3.4 GFET immunosensor assembly and characterization

**Fig. 4:**
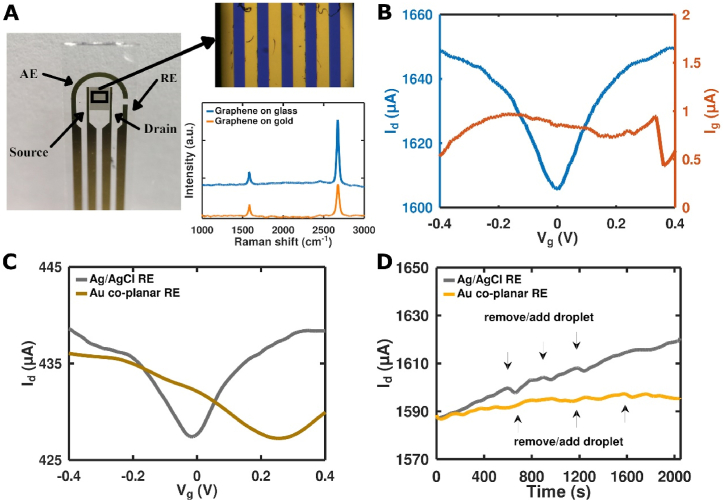
Characterization of GFETs. **(A)** Photo of IDE chip with transferred graphene. Inset: (up) optical image of IDEs with transferred graphene, scale bar 20 μm; Raman spectra of graphene (down) on glass (blue line) and on gold (yellow line). **(B)** I_d_-V_g_ (blue) and I_g_-V_g_ curves (orange) for GFET1 with Ag/AgCl electrode, V_ds_ = 40 mV. **(C)** Comparison of I_d_-V_g/Ag/AgCl_ (grey line) and I_d_-V_g/RE_ (gold line), V_ds_ = 10 mV. **(D)** Time course stability of GFET1 in 0.1xPBS using Ag/AgCl (grey line) and Au co-planar RE (yellow line) as a liquid gate.

To achieve allergen detection with increased sensitivity, the GFET immunosensor was established using Bet v 1-specific antibodies that were immobilized on a functionalized graphene monolayer. The graphene transfer process was optimized to precisely adjust the monolayer with size 3 × 3 mm to cover the ID working electrodes (WE) (Fig. 4A) and to decrease the leakage current. Raman spectra showed very sharp G and 2D bands, with no D band, suggesting a negligible number of defects in the graphene lattice after its transfer onto the IDE surface. Moreover, the 2D/G band ratio was higher for glass-supported graphene (≈5.1) than for graphene on gold electrodes (≈2.8), which is reflected by the lower intensity of the 2D band for graphene on the gold surface. This difference can be associated with the higher roughness of the gold surface compared to the glass substrate. Nevertheless, both ratios are representative for a monolayer graphene of high quality and purity. We observed a very narrow, close to zero, Dirac point providing additional information on graphene quality after PMMA cleaning; in case of Ag/AgCl as an external gate electrode, V_Dirac_ ≈ 0 V (Fig. 4B). For the Au RE gating, we observed a Dirac point shift to a positive gate voltage (V_Dirac_ = 0.25 V) (Fig. 4C) due to a potential mismatch. It should be noted that the leakage current (I_g_) is three orders of magnitude less than working current (I_d_) allowing the sensor to be used as immersible with a co-planar RE electrode. The field-effect decreased when using the co-planar RE, with a decrease in transconductance at the linear part of the curve that may be related to the smaller capacity formed by the RE.

The time-course stability of I_d_ at V_g/Ag/AgCl_ = −0.1V (hole carrier regime), V_ds_ = 40 mV was measured for 30 min (Fig. 4D). During this time, the PBS droplet (40 μL) was removed/added three times. Then, the gate electrode was replaced by a co-planar RE and time course stability at V_g/RE_ = 0V (hole carrier regime) was measured. We observed that the current had a similar noise level for both types of gate electrodes but exhibited more stable characteristics when using a co-planar RE.

**Fig. 5:**
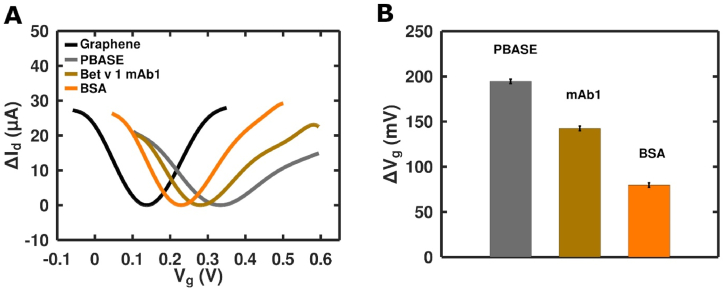
Assembly of GFET-based immunosensors. **(A)** I_d_ vs. V_g/Ag/AgCl_ during the immobilization steps on GFET1. **(B)** Shift in Dirac point measured after each step during assembly.

For the development of the biosensor, we performed deposition of PBASE, antibody and BSA. After each step of the assembly of the GFET immunosensor, the CVC curves were measured. PBASE was adsorbed by π-π staking mechanism and demonstrated a high doping effect in graphene and carbon nanotubes [15]. A noticeable p-type doping of graphene, caused by charge transfer, was observed [16], with a Dirac point shift from 130 mV to 320 mV (Fig. 5A and B). PBASE reacts with the antibody's natural amino group by forming a covalent amide bond. After antibody binding, a small but still noticeable left Dirac point shift [17] to 260 mV was observed, which is typical for antibodies. Additionally, a noticeable left shift of the Dirac point to 200 mV was observed after the blocking process using BSA.

**Fig. 6:**
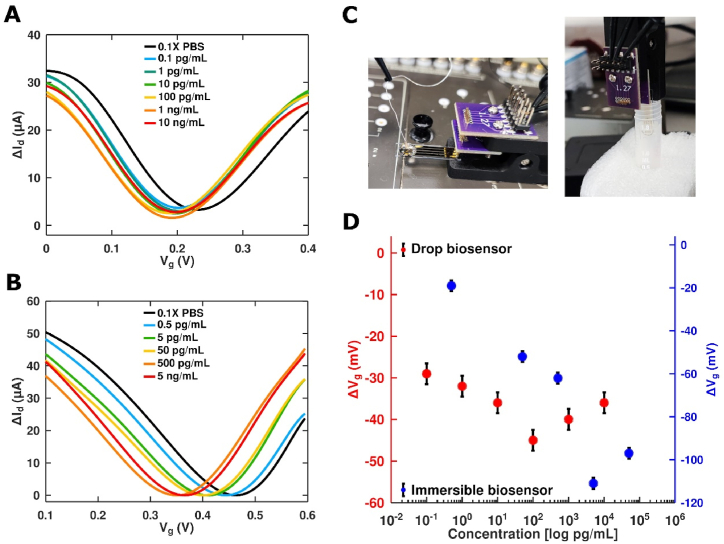
Bet v 1 analysis in 0.1xPBS using GFET immunosensor. **(A)** I_d_-V_g/Ag/AgCl_ curves at different concentrations of Bet v 1. **(B)** I_d_ - V_g/AE_ curves for different concentrations of Bet v 1. **(C)** Experiment on GFET1 with drop of analyte (left) and GFET2 immersed in analyte (right). **(D)** Calibration curve for Dirac point shift vs. Bet v 1 concentration for drop-based () and immersible () measurements of GFET biosensors.

The prepared solutions of Bet v 1 in 0.1xPBS were used to determine the GFET biosensor's performance. The results of drop-based measurements for GFET1 (Fig. 6A) demonstrated a weak left shift of the Dirac point starting from the lower concentration. Nevertheless, the saturation of signal is observed at 0.1 ng/mL with an opposite shift of the Dirac point likely due to non-specific accumulation of proteins. GFET2 was used as an immersible probe for real-time detection of different concentrations of Bet v 1 in solutions (Fig. 6B). The probe was sequentially immersed in a reaction tube with additionally added 800 μl of 0.1x PBS +0.05 % Tween and I_d_-V_g/RE_ was measured at V_ds_ = 40 mV. We also observed a left shift of the Dirac point that was more noticeable, reproducible and accurate. Experimental setups for drop-based and immersible biosensing are presented in Fig. 6C.

**Fig. 7:**
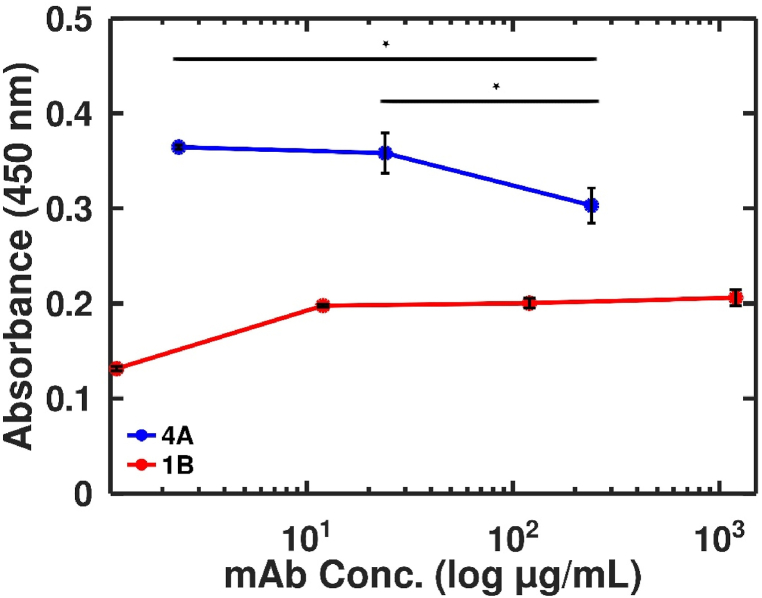
Effect of monoclonal antibody oversaturation on the sensitivity. Titration of mAb2 leads to a significant improvement of sensitivity to detect Bet v 1. For statistical analysis, a one-way ANOVA was used.

The data for the Dirac point shift were compared for two sensors (Fig. 6D). With increasing concentrations, we observed a linear logarithmic increase in the response of both biosensors. The magnitude of the sensor response was higher in case of the immersible biosensor compared to the drop-based biosensor shown in Fig. 6A and B. The GFET1 biosensor exhibited saturation at 100 pg/mL, while the GFET2 signal saturated at concentrations higher than 4 ng/mL. The LOD of GFET2 sensor was less than 10 pg/mL and overcame the performance of the other two traditional methods used in this work (Table 1).

Since pure mAbs were coupled to the biosensors without further dilution, we wanted to test if oversaturation might influence the antibody binding activity in ELISA (Fig. 7). The ELISA data showed a significant increase in sensitivity of 18–20 % when diluting mAb2 1:10 or 1:100. These data indicate that the LOD of the biosensor could be further improved by coupling lower mAb concentrations onto it. For mAb1, the undiluted antibody reached the upper plateau and further decreased with dilution.

**Fig. 8:**
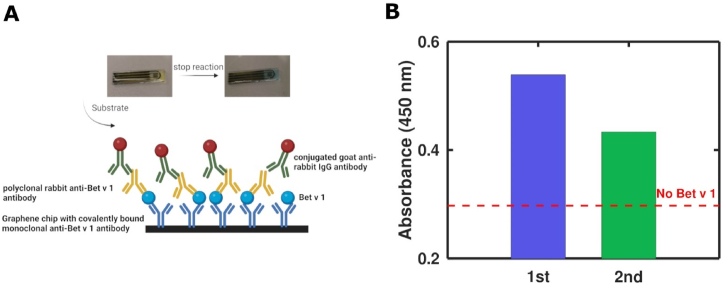
Test for reusability of the GFET biosensor coated with mAb2. **(A)** Principle of the experimental setup. **(B)** Repeated measurement of the biosensor, with a renaturation step after each run. No antigen control performed in between the two measurements is shown as a dotted line.

To further confirm proper functionality of the immobilized mAbs and the biosensor, and to address the possibility of reusing the GFET, the established ELISA protocol was performed using the GFET2 biosensor as the assay platform (Fig. 8). A Bet v 1 concentration of 1 μg/mL yielded an absorbance value of OD = 0.539 in the first run and dropped by 20 % (OD = 0.433) in the second run. The signal of the no-antigen control was always lower (OD = 0.297). These data further prove that the mAbs immobilized on the graphene platform are functionally active and indicate that reuse is possible but results in a decreased assay performance.

## 4 Discussion

Tracking of air pollen counts is still a gold standard method for monitoring the pollen season and to predict its risk for allergic patients. However, pollen traps only capture the pollen but do not provide further information on the allergen content. The relationship between pollen levels and allergic symptoms are often inconclusive, and allergens might still be very high in comparison to pollen counts. Weather extremes including heavy rain or thunderstorms can lead to the burst of pollen grains, releasing particles into the atmosphere that carry airborne allergens. These particles can further reach lower airways leading to strong asthma symptoms [18]. Recently, there are numerous articles available stating the need of a combined use of pollen counts, allergenic load, air pollution and meteorological data, which altogether provide crucial information for the management of pollen allergies. Topographical data on pollen, such as using Hysplit models, are highly important since long-distance traveling of pollen due to their optimal morphology (e.g., Betula or Olea) should also be considered. Measured pollen levels and allergen concentrations do not necessarily result from local trees, and selective pruning of such trees, as suggested, is not effective. To still avoid the spreading of locally produced pollen, training of municipal officials is very relevant to avoid the planting of new anemophilous species in cities [7,[19], [20], [21]]. There is an urgent need to improve the resolution of pollen data, which can be partially achieved by using artificial intelligence [3] and incomplete pollen monitoring networks need to be expanded [22].

The most common method for allergen detection, derived from different allergenic sources like pollen, house dust or animal dander is the ELISA. However, this approach faces several disadvantages, such as the requirement of proper lab equipment together with a long assay time (1–2 days). Moreover, the biggest disadvantage of this detection method is its low sensitivity as shown in our experiments. It is very likely that a higher sampling flow rate would lead to sufficient quantification of Bet v 1 as shown elsewhere [23]. Newer approaches, like multiplex bead-based assays for airborne and indoor allergen detection, recognizing several allergens in parallel, reaching better detection ranges but still require proper lab equipment and skilled personnel and are not applicable for field measurements [24]. As our data showed, our established mediator release assay represents a highly sensitive and robust method for allergen measurement that can detect air sampled pollen even in small quantities. Like the other above-mentioned approaches, appropriate instruments and knowhow are needed and are not suitable for real-time measurements.

Here, a GFET transistor was developed on a commercial IDE for highly sensitive immunosensing of Bet v 1. Upon the functionalization of graphene, a shift in the Dirac point was monitored as a confirmation of its surface modification. The reproducible trend for both, mAb1 as the receptor and BSA as the blocking agent, suggests that proteins bring a larger positive charge, which is supported by literature [25]. Though proteins can bring a positive charge when the isoelectric point is higher than the pH of the solution (7.4), the main effect is explained by an increase of the local Debye length due to the protein's own electrical double layer (EDL) [26]. This not only can lead to a Dirac point shift but also to a decrease of the field effect, i.e., a transconductance decrease. Moreover, upon Bet v 1 binding to the immobilized mAbs, a further left shift of the Dirac point indicates that Bet v 1 brings additional positive charge. Compared to other, already published graphene-based biosensors for allergen detection (Table 2), the direct electronic detection of the signal via GFET provides an accurate and potentially real-time measurement of airborne allergens for in-field measurements. This is the first time to our knowledge that allergens from airborne pollen were analyzed whereas other proposed methods focused only on the detection of food allergens. The principle of our sensor was based on the immobilization of allergen-specific monoclonal IgG antibodies whereas similar studies relied on aptamers or hybridization of stem-loop DNA with the target DNA of the allergen and did not always reach high sensitivity [[27], [28], [29]]. Such DNA-based sensors face challenges such as susceptibility to nuclease degradation and environmental sensitivity, which can impact their stability and binding affinity. Aptamers also struggle with non-specific binding and a limited dynamic range, alongside issues in sensor regeneration and consistency due to batch-to-batch variability in aptamer synthesis; all limitations we have overcome with our antibody-based approach.

**Table 2:** The Bet v 1-biosensor described herein (highlighted in grey) in comparison with other already published sensors.

Detection method	Sensing material	Allergen type	Target	Limit of detection	Detection range	Assay time	Ref.
FET	Antibody/Graphene	Airborne	Bet v 1	10 pg/mL	4 ng/mL −10 pg/mL	12 min, real-time	
DPV	Antibody/Graphene/SPE	Food	Ovalbumin	0.83 pg/mL	500 ng/mL −1 pg/mL	45 min	[8]
DPV	DNA/GO/Au NP/GCE	Food	Ara h 1	0.041 fM	100 fM−0.1 fM	N/A	[28]
FRET	QD/Aptamer/GO	Food	Ara h 1	56 ng/mL	2000 ng/mL - 56 ng/mL	10 min	[29]
SWV	Aptamer/Graphene/SPE	Food	β-lactoglobulin	20 pg/mL	100 ng/mL −20 pg/mL	20 min	[27]

FET, field-effect transistor; DPV, differential pulse voltammetry; GO, graphene oxide; GCE, glassy carbon electrode; SPE, screen printed electrodes; NP, nanoparticles; SWV, square wave voltammetry; QD, quantum dot; FRET, Förster resonance energy transfer.

Two approaches for sampling were used here, drop and immersible sensing, the former associated with placing a drop of the sample on the immunosensor and the latter immersing the immunosensor into the sample. A higher dynamic range for the immersible approach was found that can be explained by the used antibody (mAb2) for GFET2 functionalization, which has demonstrated the highest binding affinity to Bet v 1 in the ELISA experiments conducted previously.

Using the immobilization of GFET biosensors with Bet v 1-specific mAb with different affinities, we have demonstrated that in both cases the device can provide high sensitivity, while the affinity mostly affects the dynamic range. This proves the high sensitivity to environmental change of graphene as a transducing element, compared to the low sensitivity of the weak colorimetric signal in the ELISA assay. Our developed GFET-based sensor demonstrated the highest sensitivity and fastest assay time, which, in combination with its disposability and easy-to-use features makes it a highly attractive device in personal monitoring of the environment for early prediction during the pollen season. Since the biosensor is more sensitive in detecting Bet v 1 than the RBL assay, the air pollen collected over a period of 6 h (4500 L) would be enough for detection. The ventilation rate of a resting human is about 6 L per minute but can reach more than 140 L per minute upon heavy exercise [30]. With each breath, the possibility of inhaling airborne pollen, leading to allergic symptoms increases. Depending on the geographical location and environmental factors, the birch pollen count (grains/m^3^) can exceed several hundred grains during the peak pollen season, each grain containing about 3.2 pg Bet v 1 [23]. Since already a short time exposure to only a few of those grains lead to strong allergic symptoms in an allergic patient, it is of highest physiological relevance to even detect very low quantities of allergens which cannot be detected with classical approaches; especially in the context of pollen forecast systems. Further studies are needed to develop a point-of-need device for the real-time measurement of pollen allergens, combined with personal air sampling pumps already available on the market [31], which will simplify the analysis of one's individual allergen burden in the future. Such device could sample the air, extract the pollen directly in aqueous solutions (e.g. using microfluidic systems) and quantify the allergenic load with the herein described biosensor. The aqueous extraction in this process is important to release allergens from the pollen grain by hydration, an important step in germination. Major pollen allergens as Bet v 1 are highly soluble and released within a few minutes [32]. To have a general overview of the allergenic content in the air, pollen monitoring stations could use such biosensor and thereby generated data are readily transferable to the public.

## 5 Conclusion

We have developed and optimized an immersible GFET-based biosensor that is highly efficient in detecting Bet v 1 compared to commonly used ELISA and mediator release assays. By requiring a total detection time of just 12 min, this approach represents the currently fastest detection method for allergen quantification to our knowledge, which might be implemented in in-field measurement devices in the future. The combination of (i) determination of the allergen content (ii) pollen counts and (iii) updated and accurate pollen maps is important for drawing conclusions about the allergenicity risk, which helps allergic patients to start immediate interventions and will improve their quality of life.

## Ethics approval and consent to participate

Not Applicable.

## Consent for publication

Yes.

## Data availability statement

Data will be made available on request.

## CRediT authorship contribution statement

**Stefan Jarić:** Writing – original draft, Visualization, Validation, Investigation, Formal analysis. **Mario Wenger:** Writing – original draft, Visualization, Validation, Methodology, Investigation, Data curation. **Ivan Bobrinetskiy:** Writing – review & editing, Writing – original draft, Supervision, Project administration, Methodology, Funding acquisition, Conceptualization. **André Stapelfeldt:** Writing – review & editing, Investigation, Data curation. **Glorismer Pena-Amelunxen:** Writing – review & editing, Methodology, Investigation. **Branko Šikoparija:** Writing – review & editing, Supervision, Resources, Project administration, Methodology, Investigation, Funding acquisition, Conceptualization. **Lorenz Aglas:** Writing – review & editing, Writing – original draft, Supervision, Resources, Project administration, Methodology, Funding acquisition, Conceptualization.

## Declaration of competing interest

The authors declare that they have no known competing financial interests or personal relationships that could have appeared to influence the work reported in this paper.

## References

[1] Biedermann T., Winther L., Till S.J., Panzner P., Knulst A., Valovirta E. (2019). Birch pollen allergy in Europe. Allergy.

[2] Buters J., Clot B., Galán C., Gehrig R., Gilge S., Hentges F., O'Connor D., Sikoparija B., Skjoth C., Tummon F., Adams-Groom B., Antunes C.M., Bruffaerts N., Çelenk S., Crouzy B., Guillaud G., Hajkova L., Seliger A.K., Oliver G., Ribeiro H., Rodinkova V., Saarto A., Sauliene I., Sozinova O., Stjepanovic B. (2024). Automatic detection of airborne pollen: an overview. Aerobiologia.

[3] Makra L., Matyasovszky I., Tusnády G., Ziska L.H., Hess J.J., Nyúl L.G., Chapman D.S., Coviello L., Gobbi A., Jurman G., Furlanello C., Brunato M., Damialis A., Charalampopoulos A., Müller-Schärer H., Schneider N., Szabó B., Sümeghy Z., Páldy A., Magyar D., Bergmann K.C., Deák Á J., Mikó E., Thibaudon M., Oliver G., Albertini R., Bonini M., Šikoparija B., Radišić P., Josipović M.M., Gehrig R., Severova E., Shalaboda V., Stjepanović B., Ianovici N., Berger U., Seliger A.K., Rybníček O., Myszkowska D., Dąbrowska-Zapart K., Majkowska-Wojciechowska B., Weryszko-Chmielewska E., Grewling Ł., Rapiejko P., Malkiewicz M., Šaulienė I., Prykhodo O., Maleeva A., Rodinkova V., Palamarchuk O., Ščevková J., Bullock J.M. (2023). A temporally and spatially explicit, data-driven estimation of airborne ragweed pollen concentrations across Europe. Sci. Total Environ..

[4] Schäppi G.F., Suphioglu C., Taylor P.E., Knox R.B. (1997). Concentrations of the major birch tree allergen Bet v 1 in pollen and respirable fine particles in the atmosphere. J. Allergy Clin. Immunol..

[5] Taylor P.E., Jonsson H. (2004). Thunderstorm asthma. Curr. Allergy Asthma Rep..

[6] Nickovic S., Petković S., Ilić L., Pejanović G., Mijić Z., Huete A., Marks G. (2023). Prediction of airborne pollen and sub-pollen particles for thunderstorm asthma outbreaks assessment. Sci. Total Environ..

[7] Suanno C., Sandrini S., Aloisi I., De Nuntiis P., Facchini M.C., Del Duca S., Fernández-González D. (2022). Airborne pollen, allergens, and proteins: a comparative study of three sampling methods. Sustainability.

[8] Eissa S., L'Hocine L., Siaj M., Zourob M. (2013). A graphene-based label-free voltammetric immunosensor for sensitive detection of the egg allergen ovalbumin. Analyst.

[9] van Ree R., Chapman M.D., Ferreira F., Vieths S., Bryan D., Cromwell O., Villalba M., Durham S.R., Becker W.M., Aalbers M., André C., Barber D., Cistero Bahima A., Custovic A., Didierlaurent A., Dolman C., Dorpema J.W., Di Felice G., Eberhardt F., Fernandez Caldas E., Fernandez Rivas M., Fiebig H., Focke M., Fötisch K., Gadermaier G., Das R.G., Gonzalez Mancebo E., Himly M., Kinaciyan T., Knulst A.C., Kroon A.M., Lepp U., Marco F.M., Mari A., Moingeon P., Monsalve R., Neubauer A., Notten S., Ooievaar-de Heer P., Pauli G., Pini C., Purohit A., Quiralte J., Rak S., Raulf-Heimsoth M., San Miguel Moncin M.M., Simpson B., Tsay A., Vailes L., Wallner M., Weber B. (2008). The CREATE project: development of certified reference materials for allergenic products and validation of methods for their quantification. Allergy.

[10] Chapman M.D., Ferreira F., Villalba M., Cromwell O., Bryan D., Becker W.M., Fernández-Rivas M., Durham S., Vieths S., van Ree R. (2008). The European Union CREATE project: a model for international standardization of allergy diagnostics and vaccines. J. Allergy Clin. Immunol..

[11] Huber S., Lang R., Steiner M., Aglas L., Ferreira F., Wallner M., Hawranek T., Gadermaier G. (2018). Does clinical outcome of birch pollen immunotherapy relate to induction of blocking antibodies preventing IgE from allergen binding? A pilot study monitoring responses during first year of AIT. Clin. Transl. Allergy.

[12] Soh W.T., Aglas L., Mueller G.A., Gilles S., Weiss R., Scheiblhofer S., Huber S., Scheidt T., Thompson P.M., Thompson P.M., Briza P., London R.E., Traidl-Hoffmann C., Cabrele C., Brandstetter H., Ferreira F. (2019). Multiple roles of Bet v 1 ligands in allergen stabilization and modulation of endosomal protease activity. Allergy.

[13] Pointner L., Kraiem A., Thaler M., Richter F., Wenger M., Bethanis A., Klotz M., Traidl-Hoffmann C., Gilles S., Aglas L. (2021). Birch pollen induces toll-like receptor 4-dependent dendritic cell activation favoring T cell responses. Front. Allergy.

[14] Wenger M., Bethanis A., Johnson L., Aglas L. (2021). Humanized mediator release assay as a read-out for allergen potency. J. Vis. Exp..

[15] Sobhan A., Lee J., Park M.-K., Oh J.-H. (2019). Rapid detection of Yersinia enterocolitica using a single–walled carbon nanotube-based biosensor for Kimchi product. LWT.

[16] Mishyn V., Hugo A., Rodrigues T., Aspermair P., Happy H., Marques L., Hurot C., Othmen R., Bouchiat V., Boukherroub R., Knoll W., Szunerits S. (2022). The holy grail of pyrene-based surface ligands on the sensitivity of graphene-based field effect transistors. Sens. Diagn.

[17] Fernandes E., Cabral P.D., Campos R., Machado G., Cerqueira M.F., Sousa C., Freitas P.P., Borme J., Petrovykh D.Y., Alpuim P. (2019). Functionalization of single-layer graphene for immunoassays. Appl. Surf. Sci..

[18] D'Amato G., Annesi-Maesano I., Urrutia-Pereira M., Del Giacco S., Rosario Filho N.A., Chong-Neto H.J., Solé D., Ansotegui I., Cecchi L., Sanduzzi Zamparelli A., Tedeschini E., Biagioni B., Murrieta-Aguttes M., D'Amato M. (2021). *Thunderstorm allergy and asthma: state of the art.* Multidiscip. Respir. Med..

[19] Álvarez-López S., Fernández-González M., González-Fernández E., Garrido A., Rodríguez-Rajo F.J. (2020). Tree allergen pollen-related content as pollution source in the city of ourense (NW Spain). Forests.

[20] Álvarez-López S., Fernández-González M., Sánchez Espinosa K.C., Amigo R., Rodríguez-Rajo F.J. (2022). London plane tree pollen and Pla A 1 allergen concentrations assessment in urban environments. Forests.

[21] Bayr D., Plaza M.P., Gilles S., Kolek F., Leier-Wirtz V., Traidl-Hoffmann C., Damialis A. (2023). Pollen long-distance transport associated with symptoms in pollen allergics on the German Alps: an old story with a new ending?. Sci. Total Environ..

[22] Markey E., Clancy J.H., Martínez-Bracero M., Maya-Manzano J.M., Smith M., Skjøth C., Dowding P., Sarda-Estève R., Baisnée D., Donnelly A., McGillicuddy E., Sewell G., O'Connor D.J. (2022). A comprehensive aerobiological study of the airborne pollen in the Irish environment. Aerobiologia.

[23] Buters J.T.M., Thibaudon M., Smith M., Kennedy R., Rantio-Lehtimäki A., Albertini R., Reese G., Weber B., Galan C., Brandao R., Antunes C.M., Jäger S., Berger U., Celenk S., Grewling Ł., Jackowiak B., Sauliene I., Weichenmeier I., Pusch G., Sarioglu H., Ueffing M., Behrendt H., Prank M., Sofiev M., Cecchi L. (2012). Release of Bet v 1 from birch pollen from 5 European countries. Results from the HIALINE study. Atmos. Environ..

[24] Zahradnik E., Raulf M. (2014). Animal allergens and their presence in the environment. Front. Immunol..

[25] Kwon S.S., Kim D., Yun M., Son J.G., Lee S.H. (2021). The role of graphene patterning in field-effect transistor sensors to detect the tau protein for Alzheimer's disease: simplifying the immobilization process and improving the performance of graphene-based immunosensors. Biosens. Bioelectron..

[26] Kesler V., Murmann B., Soh H.T. (2020). Going beyond the Debye length: overcoming charge screening limitations in next-generation bioelectronic sensors. ACS Nano.

[27] Eissa S., Zourob M. (2017). In vitro selection of DNA aptamers targeting β-lactoglobulin and their integration in graphene-based biosensor for the detection of milk allergen. Biosens. Bioelectron..

[28] Sun X., Jia M., Guan L., Ji J., Zhang Y., Tang L., Li Z. (2015). Multilayer graphene-gold nanocomposite modified stem-loop DNA biosensor for peanut allergen-Ara h1 detection. Food Chem..

[29] Weng X., Neethirajan S. (2016). A microfluidic biosensor using graphene oxide and aptamer-functionalized quantum dots for peanut allergen detection. Biosens. Bioelectron..

[30] Pleil J.D., Ariel Geer Wallace M., Davis M.D., Matty C.M. (2021). The physics of human breathing: flow, timing, volume, and pressure parameters for normal, on-demand, and ventilator respiration. J. Breath Res..

[31] Tulum L., Deag Z., Brown M., Furniss A., Meech L., Lalljie A., Cochrane S. (2018). Airborne protein concentration: a key metric for type 1 allergy risk assessment-in home measurement challenges and considerations. Clin. Transl. Allergy.

[32] Vrtala S., Grote M., Duchêne M., van Ree R., Kraft D., Scheiner O., Valenta R. (1993). Properties of tree and grass pollen allergens: reinvestigation of the linkage between solubility and allergenicity. Int. Arch. Allergy Immunol..

